# Secretory breast carcinoma with metastatic sentinel lymph node

**DOI:** 10.1186/1477-7819-4-88

**Published:** 2006-12-06

**Authors:** Salvatore Vieni, Daniela Cabibi, Calogero Cipolla, Salvatore Fricano, Giuseppa Graceffa, Mario Adelfio Latteri

**Affiliations:** 1 Department of Oncology, Division of General and Oncological Surgery, University of Palermo, Palermo, Italy; 2 Institute of Pathology, University of Palermo, Palermo, Italy

## Abstract

**Background:**

Secretory mammary carcinoma is a rare breast neoplasia originally described in children but sometimes also found in adults. It presents a more favourable outcome than more common histological types of breast carcinoma; published literature in fact reports only a few cases with axillary lymph node metastases and only four cases with distant metastases.

**Clinical presentation:**

In this paper we report a rare case of secretory breast carcinoma with axillary lymph node metastases in a 33-year-old woman. To our knowledge, this is the first case of secretory carcinoma involving biopsy of the sentinel lymph node and investigation of the e-cadherin expression.

We found positivity for e-cadherin, which would support the hypothesis that this type of tumour is a variant of the infiltrating ductal carcinoma.

**Conclusion:**

After a careful analysis of reported data, we have come to the conclusion that the treatment of choice for patients with secretory breast carcinoma should be conservative surgery with sentinel lymph node biopsy, followed by accurate follow-up. We are of the opinion that while post-operative radiotherapy is indicated in adult patients who have undergone quadrantectomy, it should not be used in children. Although several cases of secretory carcinoma have been treated with adjuvant chemotherapy, there are still no reliable data regarding the real value of such a choice.

## Background

Secretory carcinoma was first described in 1966 by McDivitt and Stewart [1]. This is a rare breast neoplasia first identified in female children and adolescents, at the average age of nine. Neoplasias with similar features were subsequently observed in male children and young boys, in adult men and in women of all ages, with the result that at a certain point this type of tumour was no longer defined as a "juvenile carcinoma" but was called instead "secretory carcinoma", since the histological examination revealed the presence of intracytoplasmic secretory material [2-4].

It is not a particularly aggressive tumour and presents an excellent prognosis. Axillary locoregional lymph node metastases are uncommon, while only four patients have shown distant metastases [5,6]. Several authors, therefore, suggest that treatment should be as conservative and non-aggressive as possible. Nevertheless, since there are very few reports in literature regarding this rare form of breast neoplasia, therapeutic approach to it tends to be fairly flexible.

In this paper, the authors describe the case of a 33-year-old woman with a secretory breast carcinoma and a metastatic sentinel lymph node; they analyse the immunophenotype pattern with reference to a possible histogenetic hypothesis and report the most frequently used therapeutic choices, with particular emphasis on use of sentinel lymph node biopsy, as an alternative to total axillary lymphoadenectomy.

## Clinical case report

A 33-year-old woman with no family history of breast carcinoma presented with a nodule in the upper outer quadrant of the right breast wich had been discovered about four mounth previously and had gradually increased slightly in volume during the intervening period. Clinical examination showed the presence of a hard, mobile, non-painful nodule with regular margins, a smooth surface and about 1,5 cm in diameter. No clinical alterations were found either in the ipsi- or contralateral lymph nodes. Ultrasound examination of the breast showed an ovular hypoechogenic formation with regular margins and 1,5 cm in diameter, diagnosable as a fibroadenoma of the breast.

Not only for this reason, but also following the patient's specific request, core biopsy of the tumour was not performed and the patient therefore immediately underwent surgical removal of the nodule under local anaesthetic. The histopathological examination of the surgical piece showed the presence of a secretory carcinoma the lesion had no capsule, since one of the resection margins was not very clear, and showed small, glistening, mucus-like foci on the cut surface.

**Figure 1:**
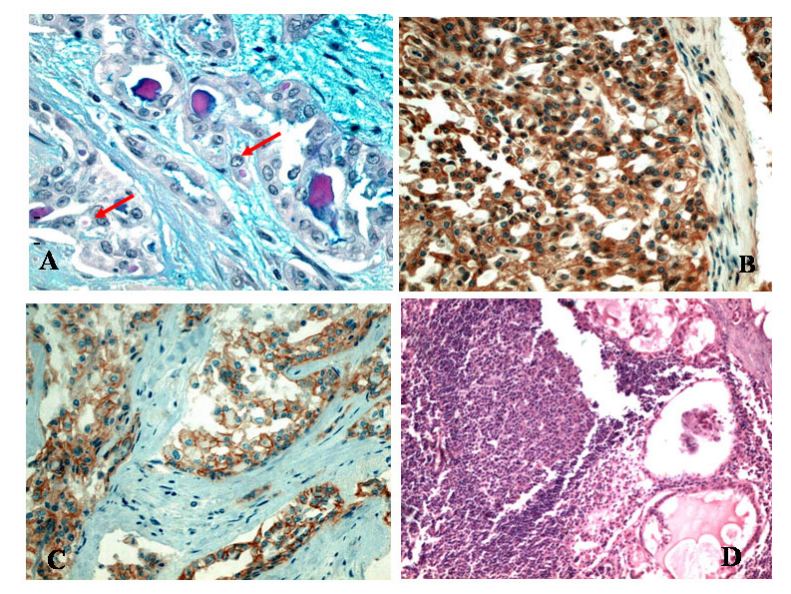
(A) PAS positive secretory material in ductular lumina and, as globular structures, in intracytoplasmic vacuoles (arrows). (B) Positive immunostain for S100 antibody, (C) Positive immunostain for E-cadherin, (D) Metastasis to sentinel lymph node. (Original magnification: A 400X; B and C 200X; D 100X).

For microscopic examination, 5 micron sections were obtained from formalin-fixed, paraffin- embedded material and stained with hematoxylin-eosin and Alcian PAS. The lesion showed solid cell nests, intermingled with tubular structures and separated by fibrous bundles. The neoplastic nests consisted of small, uniform, well-differentiated cells, with granular or vacuolated cytoplasm and vescicular nuclei containing small nucleoli. Mitoses were scanty and atypia was mild. Eosinophilic, PAS-positive secretory material was frequently observed in the ductular lumina and, in the form of globular structures, in the intracytoplasmic vacuoles of many neoplastic cells (fig 1a). Peripherally, in the more ductular areas, extracellular Alcian blue-positive material (reminiscent of a mucinous carcinoma) was present. Vascular invasion was not evident. Immunohistochemically the neoplastic cells showed a strong positive stain for cytokeratins (MNF116, CK7) EMA, S100 (fig 1b) and e-cadherin (fig 1c). In addition, an extremely focal, weak stain for 34beta E12 and CD10 was observed.

Chromogranin, sinaptophysin, NSE, smooth muscle actin, p63, Her-2, oestrogen and progesterone receptors were negative, Mib1 (Ki67) and P53 were positive in less than 5% of the cells. The histological diagnosis was G1 secretory breast cancer.

Analysis of the DNA cell content of the secretory carcinoma proves to be diploid with low proliferative activity [19-23] and, in fact, in our own case, flow cytometry performed on the cell DNA (65,520 cells) also showed a diploid-type histogram (DI = 1.0) with low proliferative activity (S-phase = 4.5%).

After an interdepartmental discussion, an upper outer quadrantectomy of the right breast on the previous site of removal of the nodule was proposed to the patient, together with a biopsy of the ipsilateral axillary sentinel lymph node.

The sentinel lymph node was identified with the use of a radiocolloid and intravital stain.

The day before the operation, the sentinel lymph node was observed by means of lymphoscintigraphy after an intradermal injection into the surgical scar of human albumin (Nanocoll) bound to technetium 99 (0,2 mCi of 99 mTc in a volume di 0,4 ml). Ten minutes before the start of the operation 0.5 ml of intravital stain was injected subdermically into the same site. A portable radioisotope detector was a further help in the intraoperative identification of the sentinel lymph node, which showed up as bright blue and was therefore removable without any difficulty. Frozen section of the sentinel nodes was not performed since at the time when this case was observed (1999) the extemporary histological examination of the sentinel lymph node was not well-codified in our department and was therefore not in routine use.

The subsequent histological analysis performed on the removed quadrant showed the presence of some small areas of "in situ" secretory carcinoma, mixed with focal aspects of ductal papillomatosis with atypia of the epithelial cells.

**Figure 2:**
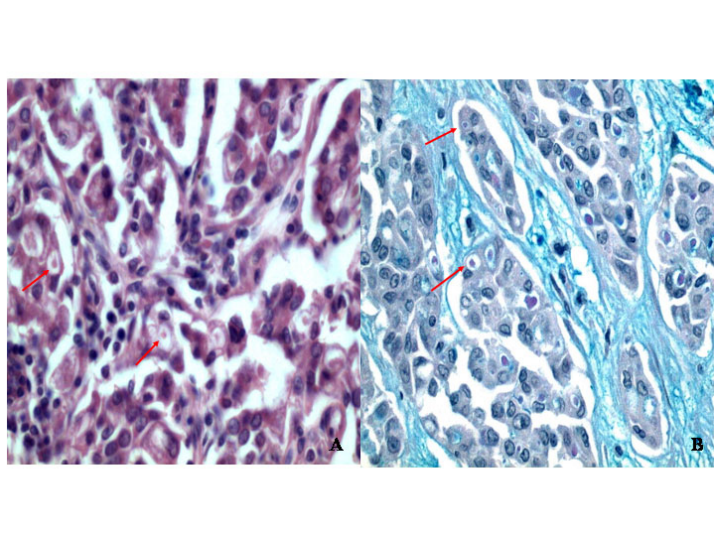
High power view of metastatic lymph node cancer cells.(A) globular structures, in intracytoplasmic vacuoles (arrows), that are positive with alcian-PAS stain (B) (Alcian)(Original magnification: A and B 400X)

The lymph node defined as the sentinel had a maximum diameter of 1 cm. It was fixed in formalin, paraffin-embedded, completely sectioned and histologically analysed. It presented metastases with a maximum diameter of 0,3 cm (fig 1d); the metastatic cancer cells showed intracytoplasmic vacuoles containing secretory globules similar to those of the primary tumour cells (Fig 2A–B)

A total axillary lymphoadectomy under general anaesthetic was therefore performed during a subsequent operation. Histological examination of the 18 axillary lymph nodes removed showed no signs of metastases. The patient was given adjuvant chemotherapy consisting of six cycles of CMF and radiotherapy of the residual mammary gland.

Periodic follow-up examinations after completion of the surgical, medical and radiotherapy treatment have shown no evidence of either local regression or distant metastases and seven years later, the patient is still free of the disease.

## Discussion

Secretory carcinoma is a rare form of breast cancer, generally with a favourable prognosis, since literature reports few cases with locoregional or distant metastases. Although this disease represents 84% of all the histological variants of infant breast carcinoma [7], secretory carcinoma can also occur in adults, both in men and women. The tumour has been found in patients ranging from three to eighty-seven years of age [1,8,9]. Most of the cases reported in literature involve female patients of the average age of 40 years and a median of 33 [10]. Only 15 cases have been observed in males (average age 17) with a male:female ratio of 1:6, which is relatively high compared with the distribution of other histological types of breast carcinoma in males [11,12].

The correlation between known risk factors for breast carcinoma and secretory carcinoma is not yet fully understood. Since the presence of oestrogen and progesterone receptors is extremely variable in patients with secretory breast carcinoma, the etiopathogenesis of such tumours is probably not linked to female sex hormones.

Many authors have shown the presence of several chromosomic anomalies in secretory carcinomas, although the significance of such genetic alterations has still to be clarified [7,10,13]. Our own case showed the two more distinctive pathological characteristics of such tumours: intracellular and extracellular secretion and granular eosinophilic cytoplasm of the neoplastic cells.

Immunohistochemically the negative stain for oestrogen and progesterone receptors and the positive stain for S100 are in agreement with previous reports [9,14,15].

The primary tumour showed a low risk factor (diameter < 2 cm, low histological grade, absence of vascular invasion, low proliferation rate assessed by Ki67, Her-2 negative immunostain), with the exception of the hormone receptors, which proved to be negative.

In our case we found strong membranous immunostain for e-cadherin. Previous studies reported that e-cadherin is present in infiltranting ductal carcinoma and its loss has been reported in most of infiltranting lobular carcinoma [16,17]. This supports the hypothesis that the secretory carcinoma might originate from the ductal component of the mammary gland and might therefore be considered as a variant of ductal carcinoma.

To our knowledge, this is the first report of secretory carcinoma in which the e-cadherin expression has been investigated. On the other hand, positivity for S100 and, focally, for CD10 and negativity for oestrogens and progesterone suggests that the tumour might originate in the basal cells of the duct, whose immunophenotype has recently been described by Kesse-Adu R and Shousha S [18]. If this view is correct, oestrogen and progesterone negativity should not be considered as the indication of an unfavourable outcome, but merely as a feature linked to the histogenetic origin of the tumour. The analysis of the DNA cell content of the secretory carcinoma proves to be diploid with a low proliferative activity [19-23]. In our case, flow cytometry performed on the cell DNA (65,520 cells) also showed a diploid-type histogram (DI = 1.0) with a low proliferative activity (S-phase = 4.5%).

Whatever the age or sex of the patient, the secretory carcinoma can usually be felt at palpation. Reported dimensions of the tumour range from a minimum of 1 cm to a maximum of 16 cm [2,21].

In agreement with reports published by several other authors [24], the tumour observed by us also presented clinical and ultrasound features similar to those of a fibroadenoma – clear margins, smooth surface and mobile on both the upper and the lower levels. Partly as a result of the clinical and echographic features of the nodule, and also following the specific request of the patient, who preferred to undergo nodulectomy only, we did not perform a core biopsy of the nodule, which would most certainly have led to a more accurate therapeutic approach and made it possible to avoid the second surgical intervention.

Breast X-rays have rarely been performed in patients with secretory carcinomas; in a multicentre study, Richard et al. described a few cases where there was radio-opacity with defined margins and the presence of microcalcifications [23].

Since this type of tumour is extremely rare, there is still no unanimous consensus about treatment choice. Many authors maintain that the frequency of local disease relapse after the simple surgical removal of the neoplasia only would suggest that mastectomy should always be performed [8,14].

According to Richard et al [23] about 33% of adult women treated with extensive surgery of the tumour site presented local disease relapse.

Whenever possible, prepubertal girls should be treated initially by wide local excision. Preservation of the breast bud should be attempted, but as this is not always possible, breast development may sometimes be impaired [14].

When the neoplasia is large, mastectomy should be performed in order to avoid the probability of local relapse [9].

Post-operative radiotherapy should be proposed after conservative surgery in adult patients, but is not advised for children because of the possible secondary effects such as fibrosis of the lung, rib damage and the consequent asymmetry of the rib cage [15,25]. In our own case, since the patient was an adult woman with a tumour of less than 2 cm, and since the histological examination of one of the nodule resection margins was not sufficiently clear, we decided on quadrantectomy followed by subsequent radiotherapy.

Although locoregional and distant metastases from the secretory carcinoma are extremely rare, they are nevertheless possible. For the correct staging of the disease, therefore, it is necessary to verify the possible presence of sytemic and/or axillary lymph node metastases.

There is still considerable discussion about the indications for a complete axillary lymph node dissection. Since the secretory carcinoma has a reduced metastatic capacity, it is obvious that a total axillary lymphoadenectomy performed on principle would be in fact a form of over-treatment for this particular disease stage. The frequently-reported post-operative complications of axillary dissection, such as pain, parasthesia, seroma, difficulty of shoulder movements and lymphedema of the upper arm are all further valid reasons for a cautious approach towards this therapeutic choice.

In the last few years, in order to avoid where possible the complete dissection of the axillary fossa in ductal and lobular breast cancer, a biopsy of the sentinel lymph node is usually performed. In our own case we also tried to use this technique, which had never been reported in literature at the time of surgery (1999). We used both intravital stain and a radiotracer with a portable radioisotope detector for the intraoperative identification and subsequent removal of the lymph node without any difficulty, even though these substances were injected directly into the surgical scar.

The histological examination of the sentinel lymph node was performed on formalin-fixed, paraffin-embedded material and showed the presence of metastases; this made it imperative to perform further surgery for total axillary lymphoadectomy. Reported data show that in 40% of cases, the other lymph nodes removed after sentinel lymph node positivity have not undergone metastatic colonisation [26] and this proved to be true in our patient.

Although several cases of secretory carcinoma have been treated with adjuvant chemotherapy, there are no published data about the real effectiveness of this therapeutic choice. Herz et al have reported non-responsiveness of the tumour to chemotherapy [6]. In our own case, since we had found axillary metastases, we administered adjuvant chemotherapy consisting of six cycles of CMF.

## Conclusion

Since very few cases of secretory carcinoma have been described in literature, it is imperative to report any new cases observed in order to establish the most suitable therapeutic approach. In this report the hypothesis that secretory carcinoma might be considered a variant of ductal carcinoma has been stressed, based on e-cadherin expression, suggesting that it might originate from the ductal component of the mammary gland. Moreover, we hypothesize that oestrogen- and progesterone-negative immunostain should not be considered as markers of an unfavourable outcome but, together with S100 and Cd10 positive immunostain, might be linked to the histogenetic origin of the tumour from the basal cells of the duct.

The fact that this neoplasia is not very aggressive suggests that surgical treatment should be kept as conservative as possible. Nevertheless, the lack of secure data regarding the real potential of the secretory carcinoma to bring about local relapse leads us to the conclusion that a conventional, conservative approach, such as quadrantectomy followed by radiotherapy, used for all other types of infiltrating breast carcinomas, should also be used for this type of tumour. Furthermore, since reports in literature have shown optimal results regarding the value of bioptic staging of the sentinel lymph gland, we are therefore of the opinion that this treatment choice is particularly valid in order to avoid axillary lymphadenectomy, useless in patients affected by neoplasias which are not particularly aggressive, such as the secretory breast carcinoma.

## Competing interests

The author(s) declare that they have no competing interests.

## Authors' contributions

**SV **and **CC **carried out the surgical procedure and contributed to the design of the study. **SF **and **GG **gathered the data from the literature search and made the first draft of the manuscript. **DC **performed the histological analysis of the surgical specimen and provided histological sections as figures for the manuscript. **MAL, SV and DC **revised and finally approved the manuscript for been published. All authors approved the final manuscript.
